# There is no general use of thromboprophylaxis and prolonged antibiotic prophylaxis in anterior cruciate ligament reconstruction: a nation-wide survey of ACL surgeons in Sweden

**DOI:** 10.1007/s00167-020-05851-7

**Published:** 2020-02-05

**Authors:** Victor Ekdahl, Anders Stålman, Magnus Forssblad, Kristian Samuelsson, Gunnar Edman, Jesper Kraus Schmitz

**Affiliations:** 1 Karolinska Institutet, Stockholm, Sweden; 2 Stockholm Sports Trauma Research Center, Department of Molecular Medicine and Surgery, Karolinska Institutet, Stockholm, Sweden; 3 Capio Artro Clinic, Stockholm, Sweden; 4 Department of Orthopaedics, Institute of Clincial Sciences, University of Gothenburg, Gothenburg, Sweden; 5 Department of Orthopaedics, Sahlgrenska University Hospital, Gothenburg, Sweden; 6 Department of Orthopaedics, Skåne University Hospital, Malmö, Sweden

**Keywords:** ACL reconstruction, Thromboprophylaxis, Antibiotic prophylaxis

## Abstract

**Purpose:**

The use of prophylaxis for thromboembolism and infection in anterior cruciate ligament (ACL) reconstruction is not well documented and no general guidelines have been established. The aim of this study was to evaluate the ACL surgeons’ individual strategies of thromboprophylaxis, use of prolonged antibiotic prophylaxis and vancomycin-soaked ACL grafts, and if its use is supported in the current literature. Additionally, the rationale for use of tourniquet was analysed.

**Methods:**

Questionnaires were distributed to all Swedish ACL surgeons who are registered in the Swedish Knee Ligament Register (SKLR), asking about prescription of thromboprophylaxis, prolonged antibiotic prophylaxis, the use of vancomycin-soaked graft and the use of a tourniquet during surgery. The responses were assessed for agreement and the thromboprophylaxis data were analysed in relation to the 2016 SKLR data.

**Results:**

115 (75%) ACL surgeons responded to the survey. 81.7% prescribed thromboprophylaxis only when risk factors, such as history of thrombosis and the use of oral contraceptives, were present. Female gender, older age and admitted patient were considered the risk factors with the lowest impact. The respondents were generally restrictive regarding the use of prolonged antibiotic prophylaxis. The use of vancomycin-soaked graft was used by only nine (8%) surgeons representing 406 (13%) of the surgeries.

**Conclusion:**

Swedish ACL surgeons are generally restrictive using thromboprophylaxis and only when risk factors are present. However, there is a lack of consensus in how to weigh the different risk factors and it does not completely adhere to the existing literature*.* Prolonged antibiotic prophylaxis is rarely used and the use of vancomycin soaking of graft is very limited and applies only to a small number of surgeons. The use of tourniquet is common. There is a need for ACL-specific guidelines regarding the use of thromboprophylaxis.

**Level of evidence:**

IV.

**Electronic supplementary material:**

The online version of this article (10.1007/s00167-020-05851-7) contains supplementary material, which is available to authorized users.

## Introduction

The value of the use of thromboprophylaxis following anterior cruciate ligament (ACL) reconstruction is poorly documented. According to American guidelines, thromboprophylaxis is not recommended in arthroscopic surgery, unless the patient has a history of venous thromboembolism (VTE) [1]. In Sweden, there are no specific guidelines on when to use thromboprophylaxis in ACL reconstruction, leaving the ACL surgeon without guidance. The negative consequences of a deep vein thrombosis (DVT) are well known and include post-thrombotic syndrome, pulmonary embolism and in worst cases cardiac arrest and death. The use of thromboprophylaxis is not without risk and its negative side effects must be taken into account. Bleeding adverse events following below-knee surgery is reported with a statistically significant OR of 2.79 comparing a group receiving thromboprophylaxis with a group with no prophylaxis [2].

The literature is sparse regarding the use of a tourniquet during ACL surgery; however, some evidence suggest it increases the risk of VTE [3]. Antibiotic prophylaxis is commonly used as a single dose in ACL surgery [4] with support in the current literature [5]. When the use of antibiotic extends more than 24 h after surgery, it is referred to as prolonged antibiotic prophylaxis, which has weak scientific evidence [6]. A novel method considered to minimize postoperative infection is to soak the graft with vancomycin [7, 8]. The use of antibiotics is not without controversy; it can lower the risk of infection for the individual patient, whereas overuse can cause bacteria to develop resistance rendering the antibiotic useless, which is major concern for the future treatment of infections [9].

The aim of this study was to evaluate the ACL surgeons’ individual strategies of thromboprophylaxis and if they correspond to existing knowledge regarding the risk factors of VTE. A secondary aim was to analyse the use of tourniquet, prolonged antibiotic prophylaxis and vancomycin-soaked ACL grafts.

## Materials and methods

The study was approved by the Regional Ethics Committee at Karolinska Institutet (2011/337-31/3).

A retrospective, cross-sectional study on the population of ACL surgeons in Sweden, was analysed and related to descriptive epidemiological register data, obtained through the Swedish Knee Ligament Register (SKLR). The SKLR, established 2005, consists of perioperative notes by the surgeon and self-reported questionnaires by the patient [4].

**Fig. 1:**
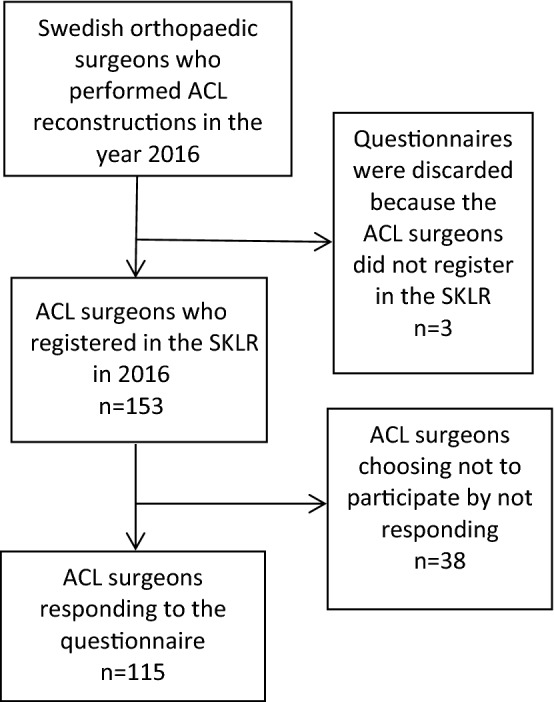
Flowchart of the cross-sectional survey aspect of the study

To get representative data applicable to the population, a response frequency of more than 70% was required, representing more than 80% of the surgeries performed 2016. Included in this study were all ACL surgeons who performed ACL reconstructions during the year of 2016 and registered their operations in the SKLR. Excluded from the study were the surgeons, who either did not respond to the survey in the given time or chose not to register in the SKLR (Fig. 1).

### Survey data

Questionnaires were distributed to all Swedish ACL surgeons who were registered in the SKLR. A paper printed version of the questionnaires was handed out during an annual Swedish conference about cruciate ligament reconstruction, May 2017. An electronic version of the questionnaire was created using an online questionnaire service (www.surveymonkey.com) and distributed with an e-mail to those surgeons not attending the national conference. The questionnaires contained questions asking if the surgeon prescribed thromboprophylaxis or prolonged antibiotic prophylaxis to minimize the postoperative complications following ACL reconstruction (ESM appendix 1). The questions were of multiple-choice nature and specifically urged the respondents to specify under what condition they used prophylaxis and if he or she used them in certain situations. Herein, the options were if the respondent “Always”, “Never” or “Under certain circumstances” prescribed prophylaxis. Should the respondent mark “Under certain circumstances”, the next question was a list of risk factors and options ranging from if these factors had “No importance”, “Some importance” “Very important” or “Decisive” to the decision of the respondent. The provided risk factors were “Older age”, “Overweight”, “Smoking”, “Duration of operation > 90 min”, “Simultaneous meniscal repair”, “Admitted patient” and “Revision surgery”. For thromboprophylaxis, the provided risk factors were “Female”, “Progestin only contraceptive pill”, “Combined oral contraceptive pill”, “History of thrombosis”, “Family history of thrombosis” and “Orthotics treatment”. To identify ACL surgeons who had not given much thought to prophylaxis, the control factor “Policy of the clinic” was added. The questionnaires also contained a varia section asking if the surgeons use tourniquet or vancomycin-soaked graft and how many ACL reconstructions they perform per year. Upon receiving questionnaires from 75.2% of the registering ACL surgeons (*n* = 115) covering 81.4% of the ACL reconstructions conducted in 2016, the survey was closed for further respondents. Three paper printed questionnaires had to be discarded since the respondents did not register in the SKLR.

Each name and acting clinic were replaced by an identification number. If the respondent completed the questionnaire twice (*n* = 3), the latest response was included in the study. In case the respondent had answered that he or she always or never prescribes prophylaxis and later checked in varying importance of different risk factors, the answer was interpreted as “Under certain circumstances”. The analysis was done for each risk factor separately; thus if the respondent chose to give prophylaxis to patients with several risk factors of “Some importance” (specified in *n* = 3), the individual factors listed were still coded “Some importance” in the agreement calculation. Therefore, only factors of independent impact on prescription was defined as agreement for prophylaxis usage.

Correlation analysis was conducted between the answers and the number of ACL surgeries performed by each respondent. To further examine if the ACL surgeons consider tourniquet as a risk for VTE needing thromboprophylaxis, another correlation analysis was made, comparing tourniquet use and thromboprophylaxis prescription. Subsequently, to evaluate if the users of the vancomycin technique prescribed less prolonged antibiotic prophylaxis to a greater extent, a cross table was constructed.

### Descriptive epidemiological register data

For external validation of the responses, information about given prophylaxis matched by risk factors was collected and analysed to ensure that the response matched the clinical use. The retrospective epidemiological register data were provided using the SKLR 2016 register data. Thus, the surgeries were conducted by the responding ACL surgeons.

Thromboprophylaxis is information registered by each ACL surgeon in the SKLR. Prolonged antibiotic prophylaxis and the use of tourniquet are not registered in the SKLR. Factors and demographics included in the retrieved 2016 register data were gender, smoking, mean age at surgery, BMI, primary or revision surgery, simultaneous meniscal suture, outpatient/inpatient, duration of operation and the choice of graft.

### Statistical analysis

Statistical analysis was conducted in SPSS statistics 25.0. The questionnaire responses were at an ordinal scale and not normal distributed and were summarized and analysed with non-parametric statistics such as frequency (*n*) and percentage (%). Demographics of the study population were also summarized using non-parametric statistics. Additional correlations of interest, such as the correlation between tourniquet use and thromboprophylaxis was calculated using Kendall’s τ-B. Differences in distribution of thromboprophylaxis according to risk factors in the 2016 SKLR register data were calculated applying the *χ*^2^ test. Statistical significance was defined as probability value *p* ≤ 0.05.

## Results

The 115 responding ACL surgeons collectively performed 3172 ACL reconstructions, including both primary and revision surgery. This corresponds to 81% [95% confidence interval (CI) 75–89%] of the registered operations during 2016. The responses represent both genders and the response rates were high, regardless of surgery volume (Table 1).

**Table 1:** The study group ACL surgeons in Sweden 2016 and the respondents of the survey

	Group	Population	Respondents
ACL surgeons, *n* (%)		153	115 (75)
	Male	137	102 (75)
	Female	16	13 (81)
ACLR, *n* (%)		3889	3172 (81.4)
ACLR per surgeon, *n* (%)	< 10	44	30 (68)
	10–30	54	40 (74)
	> 30	55	45 (82)
ACLR mean		25.4	27.6
ACLR median (range)		17 (1–103)	19 (1–103)

*ACLR* ACL reconstruction

### Thromboprophylaxis

**Fig. 2:**
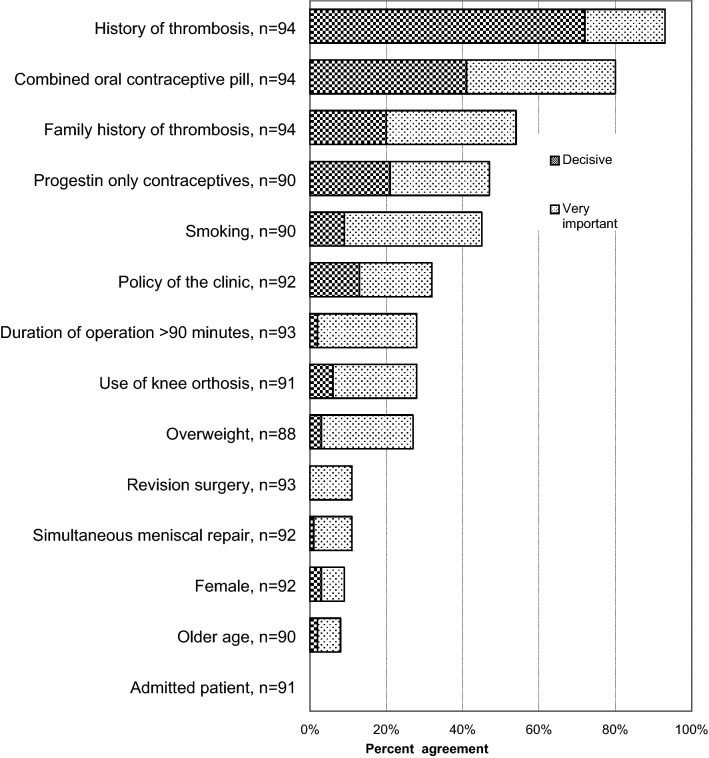
Agreement on what factors affect the ACL surgeons’ use of thromboprophylaxis. Number of respondents to this question 94. *n* = number of respondents to each statement

Out of the 115 respondents, 16% (*n* = 18) always and 3% (*n* = 3) never prescribe thromboprophylaxis, leaving 82% [*n* = 94; 95% (CI) 75–89%] to prescribe according to certain risk factors such as history of thrombosis (99%) and oral contraceptives (82%). The factor with the lowest effect was older age (9%). Admitted patient had no clinically significant importance to any surgeon’s usage of thromboprophylaxis as compared to outpatient surgery (Fig. 2).

In the “others” section for thromboprophylaxis, three additional factors were mentioned specifically by the respondents, “History of PE”, “Activated Protein C (APC)-resistance and “Long itinerary”. Furthermore, three respondents added in the comment field that their decision was governed by the number of risk factors (2 or 3 required) rather than the significance of a specific factor.

### Prolonged antibiotic prophylaxis

**Fig. 3:**
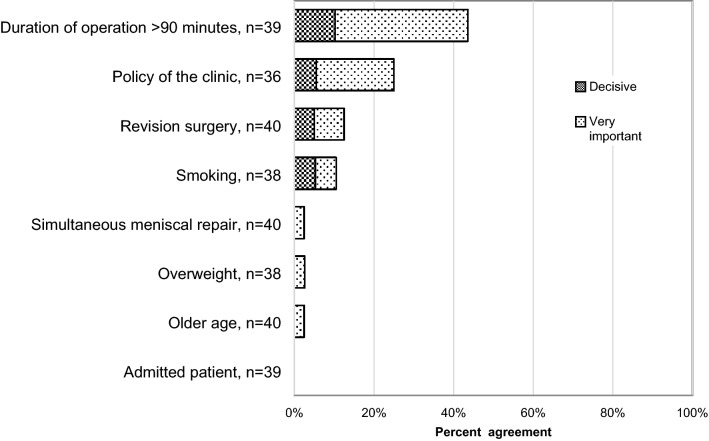
Agreement on what factors affect ACL surgeons’ use of prolonged antibiotic prophylaxis. Number of respondents to this question 44. *n* = number of respondents to each statement

The respondents were more restrictive concerning prolonged prophylaxis with 58% (*n* = 67) never prescribing prolonged antibiotic prophylaxis and 3% (*n* = 3) always doing so. This leaves 38% (*n* = 44) to risk-based assessment. No single risk factor was enough to cause prescription for ≥ 80% of the respondents. As with thromboprophylaxis, the fact that the patient was hospital admitted did not determine a single surgeon’s decision (Fig. 3). The respondents added in the “others” section several risk factors: “dermatological diseases, like acne”, “history of septic arthritis”, “allograft”, “major procedure”, “compromised sterility during the operation”, “corticosteroids or other immunosuppressive treatments”, “multi-ligament reconstruction with open wounds”, “diabetes”, and “comorbidity”.

### Surgical volume

The correlation between decisions and surgical experience was weak, suggesting that the decision was largely unaffected by how many ACL reconstructions the ACL surgeon performed. The only correlation showing statistical significance between the number of surgeries 2016 and the surgeons’ decision for thromboprophylaxis was the use of knee orthosis (CC 0.18). When it comes to prolonged antibiotic prophylaxis the more experienced, ACL surgeons were more prone to use it in cases of simultaneous meniscal suture (CC 0.25).

### Register data

In the register data, 1105 (28.6%) out of 3864 surgeries were prescribed thromboprophylaxis. Statistical significance was found for female sex (*p* < 0.001), revision surgery (*p* = 0.021), hospital admission (*p* < 0.001), duration of the operation (*p* < 0.001), and choice of graft (*p* = 0.002) (Table 2).

**Table 2:** Prescribed thromboprophylaxis: SKLR register data from 2016

		Thromboprophylaxis *n* = 1105	No thromboprophylaxis *n* = 2759	*p* value
Patient demographics				
Female, *n* (%)		585 (52.9)	1202 (43.6)	< 0.001
Male, *n* (%)		520 (47.1)	1557 (56.4)	
Smoker, *n* (%)		15 (6.0)	40 (4.5)	n.s
Non-smoker, *n* (%)		235 (94.0)	840 (95.5)	
Mean age at surgery, years (range)		28.1 (12–66)	27.6 (8–65)	n.s
Normal BMI (< 25 kg/m^2^), *n* (%)		149 (91.4)	547 (91.8)	n.s
Obese BMI (≥ 30 kg/m^2^), *n* (%)		14 (8.6)	49 (8.2)	
Perioperative data				
Revision surgery, *n* (%)		102 (9.3)	196 (7.1)	0.021
Primary surgery, *n* (%)		991 (90.7)	2553 (92.9)	
Meniscal suture, *n* (%)		138 (12.5)	372 (13.4)	n.s
No meniscal suture, *n *(%)		969 (87.5)	2396 (86.6)	
Outpatient surgery, *n* (%)		885 (79.9)	2506 (90.5)	< 0.001
Inpatient surgery, *n* (%)		222 (20.1)	262 (9.5)	
Operating time, M (SD)		89.9 (37.2)	75.7 (54.2)	< 0.001
Choice of graft				
Patellar tendon, *n* (%)		111 (10.2)	230 (8.6)	0.002
Hamstring tendon, *n* (%)		899 (82.9)	2329 (86.6)	
Allograft, *n* (%)		24 (4.1)	23 (0.9)	
Other, *n* (%)		7 (0.6)	9 (0.3)	

*n.s* non-significant

### Tourniquet

Tourniquet was always used by almost half of the surgeons [*n* = 55; 48%; 95% (CI) 39–57]. 20% (*n* = 23) use it sometimes with varying frequency, leaving 30% (*n* = 35) to never use tourniquets during ACL surgery. There was no detectable correlation between tourniquet use and the number of surgeries (*τ* = 0.01), suggesting that more experienced ACL surgeons do not differ from the less experienced; neither was there any detectable relationship between thromboprophylaxis prescription and the use of tourniquet (*τ* = 0.05).

### Vancomycin soaking

82% (*n* = 94, representing 2462 surgeries) never soak the graft in vancomycin and 8% always do (*n* = 9, representing 406 surgeries), leaving 7% (*n* = 8, representing 263 surgeries) to use it sometimes. Some respondents (*n* = 7) reported an interest in start using the technique in the near future. Table 3 presents the use of prolonged antibiotic prophylaxis matched by vancomycin soaking.

**Table 3:** The use of prolonged antibiotic prophylaxis matched by vancomycin soaking

Vancomycin soaking (number of respondents)	Prolonged antibiotic prophylaxis		
	Number of respondents (%)		
	Always	Under certain circumstances	Never
Always (9)	–	7 (78)	2 (22)
Sometimes (8)	–	4 (50)	4 (50)
Never (97)	3 (3)	33 (34)	61 (63)
Total (114)	3 (3)	44 (38)	67 (58)

## Discussion

The most important findings of this study are that Swedish ACL surgeons prescribe thromboprophylaxis only when a patient has known risk factors for thrombosis, and less than 1/3 of the patients are being prescribed thromboprophylaxis. The use of prolonged antibiotic prophylaxis was overall low in the current study with duration of the operation as the most decisive factor.

In a nation-wide study in Sweden during the years 2006–2013, the incidence of VTE following ACL surgery was 0.4%, with thromboprophylaxis prescribed to 37.1% of the patients. The prescription of thromboprophylaxis did not, however, affect the incidence of VTE [10].

There are only a few studies investigating the ACL surgeons’ reasoning behind the prescription of thromboprophylaxis [11, 12]. The study by Abouali et al. focused on the use of thromboprophylaxis among orthopaedic surgeons in Canada performing routine arthroscopy. Their results regarding risk factors were similar to ours showing that history of deep vein thrombosis and the use of oral contraceptives were attributed greatest importance. The consensus for routine thromboprophylaxis in ACL reconstruction was much lower compared to the current study. However the coverage in the Canadian study was substantially lower with a response rate of 11.2% and only included one question concerning ACL reconstruction [11]. In an Italian survey study, 94% of the respondents regularly prescribed thromboprophylaxis with high consensus for the risk factors older age (53.1%) and tourniquet (25.0%) and low consensus for oestrogen therapy (25.0%) [12]. Differences in strategy depending on nation were specifically noted in a published response to the Italian study [13].

According to the literature, specific risk factors for VTE following ACL reconstruction are operating time > 90 min [14] and older age [10, 14, 15]. These factors were not highly ranked by the ACL surgeons; thus it seems that their influence on the use of thromboprophylaxis is low and may indicate a need for increased knowledge of risk factors.

The risk factors gender, smoking and outpatient status showed quite contradicting results in the questionnaire responses versus the register data. While less than 10% of the responding ACL surgeons considered gender as an important risk factor for VTE, female patients had a significantly higher rate of prescribed thromboprophylaxis. It is possible that the high prescription rate is caused by oral contraceptives. SKLR does not keep records on patients’ contraceptive medication; hence, it was not possible to adjust for their influence. Specific studies on ACL reconstructed patients have to our knowledge not been able to prove that oral contraceptives are a risk factor; however as a general risk factor, its effect on VTE is well documented with an odds ratio of users versus non-users of 3.41 [16]. Furthermore, although ranked highly among the risk factors in the questionnaire and the literature suggesting increased risk for VTE [17], smoking had no statistically significant effect on the choice for thromboprophylaxis. A possible explanation is the low number of smokers in an otherwise healthy patient group or the underreporting of smoking habits in the SKLR. The only factor less likely to affect the ACL surgeons’ decision was hospital admission which is contradicted when analysing the register data; prescribed thromboprophylaxis is statistically significant between in- and outpatient with a higher proportion in the former group.

An additional finding in the register data is that the duration of operation was significantly longer in the thromboprophylaxis group. Longer duration of operation is associated with an increased risk of VTE [14, 18]; however there are conflicting findings; in a venographic study on patients undergoing arthroscopic surgery of the knee, the duration of operation did not influence the risk of VTE [19].

It remains uncertain whether or not application of tourniquet should be considered a risk factor for thrombosis. A systematic review showed association between VTE and tourniquet application of > 2 h [20]. In the present study, the use of tourniquet had no correlation with the overall use of thromboprophylaxis in either direction. Also, no respondent mentioned tourniquet as a reason for thromboprophylaxis in their “others” section. This may indicate that the Swedish ACL surgeons do not consider the use of tourniquet as a risk for VTE and probably find the tourniquet effective to increase the visibility. Currently, the use of tourniquet is not registered in the SKLR, which makes further analysis of the matter challenging.

In the current study, the overall low use of prolonged antibiotic prophylaxis could suggest a widespread belief that a single dose prophylaxis is the best strategy to minimize the risk of postoperative septic arthritis, which adheres well to the literature where there is no support for prolonged antibiotic prophylaxis [6]. The factor with greatest influence on the ACL surgeons’ use of prolonged antibiotic prophylaxis was duration of operation. This is in accordance with literature showing an increased risk of infection with longer duration of operation [21].

The small group of ACL surgeons (*n* = 9) using vancomycin soaking was not any less likely to prescribe prolonged antibiotic prophylaxis. This could indicate that surgeons using vancomycin are more cautious about SA in general, going to greater length in prevention. The available studies on vancomycin-soaked grafts are convincing in their ability to reduce the incidence of infection, but might be susceptible to bias in the study design; they are all cohort studies with historical control groups [7, 8, 22–24]. Thus, the results would have been swayed by every improvement done in the said time period, reducing the internal validity of the studies.

One of the main strengths of this study is the high response rate of the respondents, providing a reliable estimate of the ACL surgeons’ opinion of thromboprophylaxis. Another strength is having prophylaxis data from both the SKLR and the questionnaires. This verifies that the given answers truthfully reflect the respondent’s clinical practice.

The study is limited by not having information on important risk factors of VTE such as history of thrombosis and oral contraceptives. Information regarding these risk factors would have improved the analysis of choosing thromboprophylaxis treatment.

## Conclusions

In conclusion, the results show that Swedish ACL surgeons prescribe thromboprophylaxis to less than 1/3 of the patients. The risk factors taken into account are in line with existing literature apart from older age. The use of prolonged antibiotic prophylaxis is low.

## Electronic supplementary material

Below is the link to the electronic supplementary material.

Supplementary file1 (PDF 68 kb)
