# Supplementary material for: There is no general use of thromboprophylaxis and prolonged antibiotic prophylaxis in anterior cruciate ligament reconstruction: a nation-wide survey of ACL surgeons in Sweden
Source: Knee Surg Sports Traumatol Arthrosc. 2020 Feb 5;28(8):2535–42. doi: 10.1007/s00167-020-05851-7 (PMC7429518; doi:10.1007/s00167-020-05851-7)
Supplement: Supplementary file 1 — Supplementary file1 (PDF 68 kb) [file 167_2020_5851_MOESM1_ESM.pdf]

Thromboprophylaxis

1. Do you prescribe thromboprohylaxis for ACL reconstruction (primary- and revision surgery)?

   ☐ Under certain circumstances specified in question 2.
   ☐ Always. Continue with question 3.
   ☐ Never. Continue with question 3.

2. Please grade the risk factors below in how they affect your decision to prescribe postoperative thromboprophylaxis.

	No importance	Some importance	Very important	Decisive
Older age	☐	☐	☐	☐
Female sex	☐	☐	☐	☐
Overweight	☐	☐	☐	☐
Smoking	☐	☐	☐	☐
Progestin only contraceptive pill	☐	☐	☐	☐
Combined oral contraceptive pill	☐	☐	☐	☐
History of thrombosis	☐	☐	☐	☐
Family history of thrombosis	☐	☐	☐	☐
Orthotics treatment	☐	☐	☐	☐
Duration of operation > 90 minutes	☐	☐	☐	☐
Simultaneous meniscal repair	☐	☐	☐	☐
Admitted patient	☐	☐	☐	☐
Revision surgery	☐	☐	☐	☐
Policy of the clinic	☐	☐	☐	☐
Other 1 – which<br>…………………………………………………..<br>…………………………………………………..	☐	☐	☐	☐
Other 2 – which<br>…………………………………………………..<br>…………………………………………………..	☐	☐	☐	☐

Prolonged antibiotic prophylaxis

3. Do you prescribe prolonged antibiotic prophylaxis, i.e. postoperative antibiotics for two days or more?

   ☐ Under certain circumstances specified in question 4.
   ☐ Always. Continue with question 5.
   ☐ Never. Continue with question 5.

4. Please grade the risk factors below in how they affect your decision to prescribe prolonged antibiotic prophylaxis.

	No importance	Some importance	Very important	Decisive
Older age	☐	☐	☐	☐
Overweight	☐	☐	☐	☐
Smoking	☐	☐	☐	☐
Duration of operation > 90 minutes	☐	☐	☐	☐
Simultaneous meniscal repair	☐	☐	☐	☐
Admitted patient	☐	☐	☐	☐
Revision surgery	☐	☐	☐	☐
Policy of the clinic	☐	☐	☐	☐
Other 1 – which<br>………………………………………………..<br>………………………………………………..	☐	☐	☐	☐
Other 2 – which<br>………………………………………………..<br>………………………………………………..	☐	☐	☐	☐

Varia

5. Do you use the technique of soaking the graft in Vancomycin?

☐ Always
☐ Sometimes
☐ Never

Comments____

6. Do you use tourniquet during ACL-surgery?

☐ Always. Continue to question 8.
☐ Sometimes. Continue to question 7.
☐ Never. Continue to question 8.

7. Which factors affects your decision to use tourniquet during ACL-surgery?

☐ Old habit.
☐ Only during graft harvest.
☐ To improve visibility.
☐ Other____

8. How many ACL reconstructions (primary- and revision surgery) do you perform yearly?

☐ <10
☐ 10-30
☐ >30
